# Varying selection differential throughout the climatic range of Norway spruce in Central Europe

**DOI:** 10.1111/eva.12413

**Published:** 2016-10-23

**Authors:** Stefan Kapeller, Ulf Dieckmann, Silvio Schueler

**Affiliations:** ^1^ Department of Forest GeneticsFederal Research and Training Centre for Forests, Natural Hazards and LandscapeViennaAustria; ^2^ Evolution and Ecology ProgramInternational Institute for Applied Systems AnalysisLaxenburgAustria

**Keywords:** adaptive capacity, among‐population variation, climate change, conifers, gene flow, intraspecific variation, phenotypic variation, *Picea abies*, provenance trials, within‐population variation

## Abstract

Predicting species distribution changes in global warming requires an understanding of how climatic constraints shape the genetic variation of adaptive traits and force local adaptations. To understand the genetic capacity of Norway spruce populations in Central Europe, we analyzed the variation in tree heights at the juvenile stage in common garden experiments established from the species' warm‐dry to cold‐moist distribution limits. We report the following findings: First, 47% of the total tree height variation at trial sites is attributable to the tree populations irrespective of site climate. Second, tree height variation within populations is higher at cold‐moist trial sites than at warm‐dry sites and higher within populations originating from cold‐moist habitats than from warm‐dry habitats. Third, for tree ages of 7–15 years, the variation within populations increases at cold‐moist trial sites, whereas it remains constant at warm‐dry sites. Fourth, tree height distributions are right‐skewed at cold‐moist trial sites, whereas they are nonskewed, but platykurtic at warm‐dry sites. Our results suggest that in cold environments, climatic conditions impose stronger selection and probably restrict the distribution of spruce, whereas at the warm distribution limit, the species' realized niche might rather be controlled by external drivers, for example, forest insects.

## 1 Introduction

Understanding the constraints and drivers of species' distribution ranges is a prerequisite for predicting the consequences of climate change on natural ecosystems and for managing endangered species and populations. Within the last decade, ecologists have developed a wide variety of species distribution models to understand species' climatic and migrational limitations and to analyze the impact of climate change on biodiversity, ecosystem functions, and conservation activities (Araújo, Alagador, Cabeza, Nogués‐Bravo, & Thuiller, 2011; Hanewinkel, Cullmann, Schelhaas, Nabuurs, & Zimmermann, 2012; Randin et al., 2013; Summers, Bryan, Crossman, & Meyer, 2012; Svenning & Skov, 2004; Sykes, Prentice, & Cramer, 1996; Thomas et al., 2004; Thuiller et al., 2011). Beyond the scope of immediate abiotic and biotic interactions, evolutionary biologists aim to understand which traits determine the species' genetic capacity to adapt and expand their present ranges across certain limits (Bridle & Vines, 2007; Polechova & Barton, 2015). Strong gene flow toward marginal habitats and across heterogeneous environments was found to be the major cause of restricted ranges because it results in higher genetic load and prevents local adaptation (Haldane, 1956; Kirkpatrick & Barton, 1997; Ronce & Kirkpatrick, 2001). A key determinant of the adaptive capacity of a population in a peripheral habitat and under changing environmental conditions is the genetic variation of traits related to survival, growth, and reproduction within such populations. Populations at range limits are expected to harbor lower genetic variance within populations, because they experience stronger selection than populations under optimal conditions (Kopp & Matuszewski, 2014). On the other hand, models that allow for evolving genetic variance show that a gradually changing environment with moving optima tends to increase the genetic variance as a result of an increase in rare alleles, particularly if the population size is large (Burger & Lynch, 1995; Burger, 1999; Kopp & Matuszewski, 2014). Thus, understanding whether genetic variance varies among populations throughout a species' range and how environmental constraints affect genetic variance is required for evaluating the long‐term prospects of species in times of global change.

For trees, climate conditions are among the most important determinants of species' distributions (e.g., Araújo & Pearson, 2005; Prentice et al., 1992), meaning that the patterns of population phenotypic traits and patterns of climate conditions are related (e.g., Hannerz, Sonesson, & Ekberg, 1999; Hurme, Repo, Savolainen, & Pääkkönen, 1997; Rehfeldt et al., 2002). Provenance trials, where tree populations from a wide range of the natural distribution are planted in one or more climates, have revealed that these phenotypic responses are often based on both phenotypic plasticity and local adaptation (Morgenstern, 1996). The plastic response of populations can be used to model mean trait values both with uni‐ and multivariate climate response and genecological functions (Rehfeldt, Wykoff, & Ying, 2001; Wang, Hamann, Yanchuk, O'Neill, & Aitken, 2006) and with combined universal response functions (Wang, O'Neill, & Aitken, 2010). Such models have been found to be valuable tools for estimating the effect of climate change on growth traits and tree productivity, and for improved provenance selections (Kapeller, Lexer, Geburek, & Schueler, 2012; Mátyás, 1994; Rehfeldt et al., 2002). However, the variance of the trait means and the contribution of phenotypic plasticity to trait variance have rarely been analyzed in relation to the environmental conditions.

For Norway spruce (*Picea abies* [L.] Karst.), the most widespread conifer in Central Europe, we recently analyzed the intraspecific variation in climate response on the basis of an extensive provenance test (Nather & Holzer, 1979) where populations from almost the complete climatic distribution in Central Europe were tested across an equally wide range of test environments (Kapeller et al., 2012). This provenance test provides a unique opportunity to analyze the trait plasticity and variation throughout the species' climatic range, as Norway spruce occurs naturally from approximately 300 m up to 2,000 m above the sea level. Although a significant part of populations at low elevations are considered as secondary spruce forests, there is a long history of spruce populations in Austria, dating back to a refugial population in the alpine forelands (Ravazzi, 2002; Terhürne‐Berson, 2005). Our previous analysis (Kapeller et al., 2012) focused on the relationship between trait means and climate parameters and thus on the immediate phenotypic response to climate. Based on the observed phenotypic plasticity and the genetic variation among provenance groups, we found that populations from warm and drought‐prone areas may be appropriate candidates for extended silvicultural utilization under future climate conditions. In the present study, we aim to complement the previous analysis by investigating the phenotypic variance within and among populations across the main climate factors. The objective of this study was to quantify the phenotypic variation of height growth within and among populations of Norway spruce. To account for environmental and genetic sources of phenotypic variation, we test for the relationships between height variation and the climate of both trial sites and population origin. Moreover, we study the temporal development of phenotypic variation at the juvenile stage. Finally, we explore the distribution of the potential selection differential in populations across the species climatic niche. For this purpose, we analyze the density distributions for climatically similar groups of populations and trial sites.

## 2 Materials and Methods

### 2.1 Phenotypic data: Norway spruce provenance test 1978

**Figure 1:**
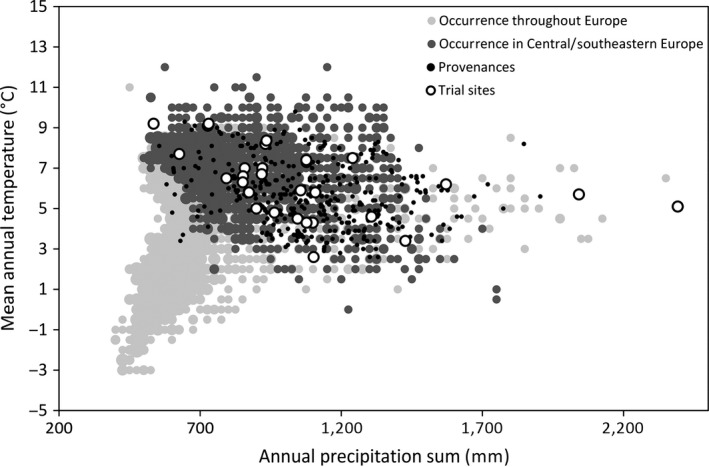
Distribution of 29 test sites (white circles) and tested populations (black dots) within the climatic range of Norway spruce. Light gray circles indicate mean annual temperature and annual precipitation of the complete Norway spruce distribution in Europe according to ICP Forests Level I monitoring plots (ICP Forests 2010). As the natural distribution in Europe can be divided into two nonoverlapping, genetically distinct ranges with different population history (Tollefsrud et al., 2008), dark gray circles indicate the central and southeastern distribution, which represent the majority of provenances

We used tree height measurements from 29 trial sites of a Norway spruce provenance test series established in 1978 in the eastern Alpine region by Nather and Holzer (1979). The original trial series comprised 44 test sites, but measurement data are available for only 29 sites. These span a wide range of altitudes from 250 to 1,750 m above the sea level; they thus comprise a large part of the climatic niche of the Norway spruce, where sites at low altitudes mark the warm and dry distribution limit and sites at high altitudes close to the tree line indicate the cold distribution limit (Fig. 1).

The seed material for the trial series was collected from 480 Austrian Norway spruce populations during commercial seed harvests in 1971. Sixty populations from other countries were also included. The Austrian harvest comprised presumably autochthonous stands and included several trees as a representative sample of the stand (Kapeller et al., 2012; Nather & Holzer, 1979). Seeds were sown over six repetitions at the central forest nursery of the Austrian Federal Forest in Arndorf (Austria) and one repetition at the experimental nursery Mariabrunn of the Austrian Federal Research and Training Centre for Forests, Natural Hazards and Landscape (BFW) in Vienna (Schulze, 1985). After 2 years, the seedlings were transferred into rows of tree nursery fields with 15 cm distance between seedlings (Schulze, 1985). In 1978, 5‐year‐old trees were transferred to the trial sites (Nather & Holzer, 1979). Each trial site was set up in a randomized complete block design with three blocks, except for sites 1 and 20, where there was only one block, and site 24, where only two blocks could be established. Because of the large number of populations sampled, not all populations could be planted at all sites. Instead, the number of tested populations per site ranged from 19 to 53 populations with an average of 28 (Table S1). The initial number of seedlings per single population per block averaged to 46.9. The seedlings of each population were planted in rectangular tree plots at a spacing of 1.5 m × 1.5 m.

The 29 trial sites were measured in 1983 and 1988, at 5 and 10 years after their establishment. This provided height data for the trees at the age of 10 and 15 years. During the 1983 measurement, the shoot length for the preceding 3 years was also measured, which provided heights for the trees at the age of 7, 8, and 9 years. Of the 109,101 trees initially planted in 1978, 83,304 could be measured in the year 1988 (on average 38.8 trees per plot). This reduction was caused by mortality, because there had been no forest management activities for the duration of the trial (1978–1988).

### 2.2 Climate data

Climate data from all the trial sites and population origins were compiled by the Austrian Central Institute for Meteorology and Geodynamics for a previous analysis (Kapeller et al., 2012). The mean climate data of trial sites strictly refer to the growing period from 1978 to 1988, whereas the climate data of population origin are based on long‐term means (1971–2008). These data include mean temperatures, mean monthly minima and maxima, and precipitation sums for both the complete year and the approximate growing season from April to September. In addition, the length of vegetation period (given as the number of days with an average temperature above 5°C), growing degree days (i.e., a thermal index accumulating degree days above a threshold of 5°C), the average day of the first frost in fall, and an annual heat moisture index according to Wang et al. (2006) were used as climate parameters.

**Figure 2:**
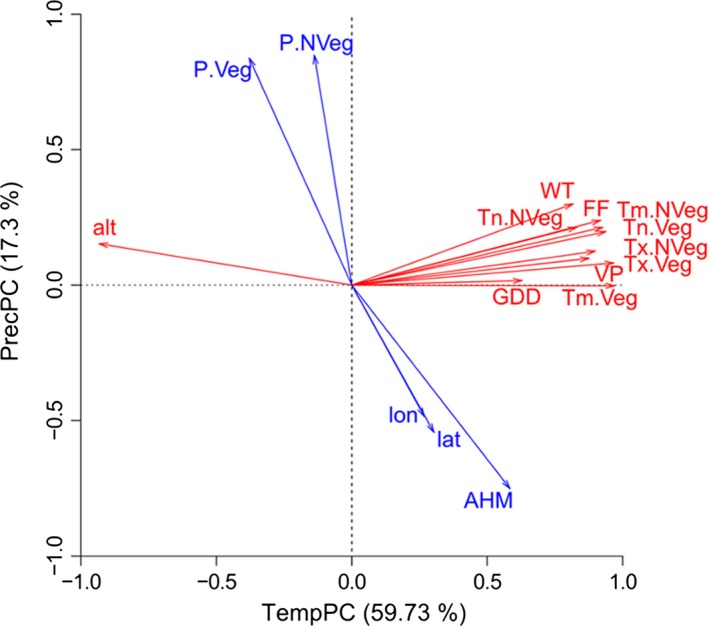
Principal component analysis of climatic parameters. The biplot shows that temperature‐related factors and altitude (red) are oriented along dimension TempPC (first principal component). Precipitation and drought index (blue) are oriented along PrecPC (second principal component). Parameters included in this analysis were temperature mean (Tm), temperature maximum (Tx), temperature minimum (Tn) and mean precipitation sums (P) during the vegetation season (.Veg) and outside the vegetation season (.NVeg), growing degree days (GDD), length of vegetation period (VP), annual heat moisture index (AHM; see Wang et al., 2006), mean winter temperature (WT), average day of first frost in fall (FF), as well as longitude (lon), latitude (lat), and altitude (alt)

To reduce the number of climate predictors and to obtain uncorrelated variables for the subsequent analysis, we performed a principal component analysis (PCA) using the statistical environment R (R Core Team 2014), with PCA functions from the R package “FactoMineR” (Husson, Josse, Le, & Mazet, 2015). The contribution of climate variables to each dimension of the PCA is given in Table S2. The first principal component explains 59.7% of observed variance and aggregates mainly temperature‐related climate parameters. The second principal component adds another 17.3% variance and aggregates mainly precipitation‐ and drought‐related parameters (Fig. 2). Therefore, we refer to the first dimension as “temperature‐related principal component” (TempPC; large values indicate warm conditions) and to the second dimension as “precipitation‐related principal component” (PrecPC; large values indicate moist conditions).

### 2.3 Statistical analyses

Our analyses were performed in four steps. First, we applied a linear model at each test site to partition among‐population variance from the total phenotypic variance. Second, we calculated the standardized within‐population variation for each population and test site and related it to the climate of the trial site and the climate of the population origin. The standardized within‐population variation of tree height was calculated for the same trees in different years at the ages of 7, 8, 9, 10, and 15 years; this was performed to assess the temporal changes in within‐population variation throughout the juvenile stage. Third, we analyzed the density distributions of tree heights with mixture‐model analysis to understand the environmental and genetic effects on phenotypic variation. In a fourth step, we validated the robustness of our results with respect to the potential biases introduced by unequal climatic distribution of tested populations across sites and varying survival rates between trial sites.

#### 2.3.1 Among‐population variation

To estimate the variance among populations from the total variance at the individual trial sites, we used a linear model in the R package “lme4” (Bates, Maechler, Bolker, & Walker, 2014). Populations and repetitions (blocks) were treated as random effects, which allowed us to extract the estimates of the among‐population variance σ²_ap_, the among‐block variance σ²_b_, and the total site variance σ²_s_ of tree heights from the fitted models. To remove the effects of different growth rates among sites and to allow for comparisons between the individual test sites, these variance components (σ²_s_, σ²_ap_, σ²_b_) were standardized as coefficients of variation using the formulas CV_s_ = σ_s_/μ_s_, CV_ap_ = σ_ap_/μ_s_, and CV_b_ = σ_b_/μ_s_, where μ_s_ is the mean tree height at the site. After calculating these variation measures for each of the 29 sites separately, the relationship of the standardized total variation CV_s_ and the standardized among‐population variation CV_ap_ to the first two principal components of site climate TempPC and PrecPC was analyzed by linear regression analysis. Additionally, the ratio of among‐population variation to the total site variation was calculated as CV_ap_/CV_s_ (which equals σ_ap_/σ_s_) to test whether the portion of explained variance by population relative to the total site variance relates to site climate. Therefore, we applied linear regression analyses using the first two PCA components of the climate parameters (TempPC and PrecPC) from the test sites as explanatory variables.

#### 2.3.2 Within‐population variation

To analyze the within‐population variation along a climatic gradient of test sites and populations, we calculated the coefficient of variation of tree heights at age 15 for each population on each site separately as CV_wp_ = σ_wp_/μ_wp_, where σ_wp_ and μ_wp_ refer to all individual tree heights measured for a specific population at a specific site. The coefficient of variation was calculated to remove the effect of different growth rates among sites. In total, we obtained 819 values of CV_wp_ for all population–site combinations. We used multiple linear regression analysis to investigate the potential relations between within‐population variation CV_wp_ and the first two PCA components of the climate parameters (TempPC and PrecPC) from the test sites as well as from the population origins. This analysis of the within‐population variation CV_wp_ was repeated with tree heights measured in earlier years, when trees aged 7, 8, 9, and 10 years, in order to identify the temporal changes in tree height variation within the juvenile stage. In addition, interactions between site and population climate were assessed in bivariate plots.

#### 2.3.3 Mixture‐model analysis

To understand the effect of site and population climate on phenotypic variation, test sites and populations were both categorized into three climatic groups. The climatic ranges of the first two PCA components (TempPC and PrecPC) were subdivided into equal intervals and populations and sites were then assigned to the corresponding climatic subgroup. The groups were labeled S1, S2, and S3 for sites and P1, P2, and P3 for populations, referring to low, medium, and high levels of TempPC or PrecPC, respectively. For low TempPC, group S1 and P1 pooled “cold” sites and populations originating from a “cold” environment, respectively, while for low PrecPC, S1 and P1 represented “dry” sites and populations originating from dry locations. The density distributions of all nine subset combinations (three population subsets X three site subsets) were plotted separately and analyzed with mixture model analysis (R package “mixtools”; Benaglia & Chauveau, 2009). Mixture model analysis provide the density probabilities of hypothetical normal‐distributed subgroups within each subset. Such probabilistic models have been used to identify subpopulations within an overall population (Benaglia & Chauveau, 2009; Ni, Baiketuerhan, Zhang, Zhao, & Von Gadow, 2014) and to test for admixture within populations using quantitative trait data. Here, we used mixture model analysis not to identify truly distinct subpopulations in a strict sense of population genetics, but to visualize the subtle patterns in the density distributions of tree heights. With our data, the mixture‐model analysis allowed us to differentiate two phenotypic subgroups of “tall” and “small” trees within each of the climatic subsets. The ratios of these subgroups provided by the mixture model analysis can be used as additional statistics to describe the shape of a density distribution.

#### 2.3.4 Robustness to stratification

**Figure 3:**
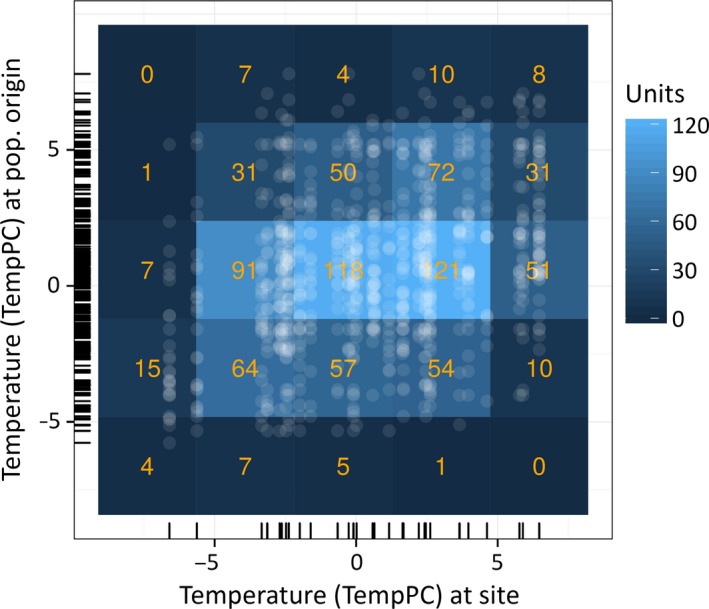
Climatic distribution of sites and populations. The bivariate climate spectrum of sites and population origins was subdivided into 25 climate strata, each representing a class of climatic site–population combinations. Relative frequency of test units, that is, site–population combinations (transparent white dots), was utilized to weight each unit and to test whether the stratification of the trial design has an effect on the outcomes of the study

The distribution of provenances to the trial sites did not follow a fully randomized procedure, but was specified in some cases according to the altitude of the test sites and populations. Thus, some populations from higher altitudes (with colder and wetter climate) were preferentially tested on (colder and wetter) sites at higher altitudes, and vice versa (see Fig. 3). As this provenance distribution had the potential to affect our analysis, we aimed to reduce the data imbalance and tested for the effects of a slightly unequal population distribution by applying two different data stratification approaches. Stratification was performed by weighting each test unit (=population × site combination) according to its relative frequency in the bivariate climate spectrum of populations and sites (Fig. 3). We divided the bivariate climate spectrum into 25 climate strata according to regular subdivisions of TempPC at sites and population origins. We then calculated weights for each unit using the relative frequency of each unit in relation to the total number of units (weight#1) or its deviation from the joint frequency probability of the populations and sites of each respective stratum (weight#2). Taking into account these weights for all population x site combinations, we reanalyzed our multiple linear regression analyses of CV_wp_.

#### 2.3.5 Effects of mortality

Of the 109,101 trees planted in 1978, 76% survived and could be measured in the year 1988 (i.e. 83,304 trees). The remaining 24% of trees died off for unknown reasons. As this mortality might also bias our estimates of height variation, we tested for the differences in the survival rate of individual populations across both the climate gradients of the trial sites and the gradient of the population origin using multiple regression analysis.

## 3 Results

### 3.1 Total tree height variation and among‐population variation are negatively related to temperature at trial sites

The mixed‐effect model yielded variance components for each site, with one variance component explained by the differences among tested populations (on average 21.8% of the total variance σ²_s_), one component explained by the differences among repetition blocks (on average 4.2% of σ²_s_), and a residual variance (on average 74% of σ²_s_). Results for each site are given in supplementary Table S1.

**Figure 4:**
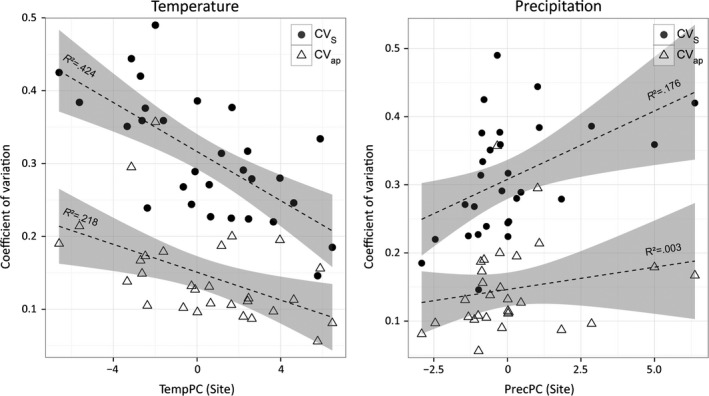
Total and among‐population variation at trial sites. Coefficients of variation at each site (CV_S_, full circles) and among‐population variation (CV_ap_, open triangles) are shown for each site along the climatic gradients of TempPC (left) and PrecPC (right). CV_S_ are high at cold‐moist sites (low TempPC, high PrecPC) and low at warm‐dry sites (high TempPC, low PrecPC)

Standardized coefficients of variation allow for comparisons among trial sites. The standardized total variation in tree heights CV_s_ ranged from 0.15 to 0.49 between the trial sites (Fig. 4, Table S1). When we partitioned the among‐population variation from the total tree height variation at each site, the standardized among‐population variation CV_ap_ ranged from 0.06 to 0.36 (Fig. 4, Table S1). The CV_ap_/CV_S_ ratio ranged across all sites from 0.25 to 0.73 and averaged 0.47.

The overall variation in tree heights CV_S_ was found to be significantly related to the climate of test sites for both TempPC (adjusted *R*² = 0.44, *p *<* *0.001) and PrecPC (adjusted *R*² = 0.19, *p* = 0.011). Here, CV_s_ was found to be high at cold‐moist sites (low TempPC, high PrecPC) and low at warm‐dry sites (high TempPC, low PrecPC). The ratio of among‐population variation (CV_ap_) to the overall variation (CV_ap_ / CV_s_) was not significantly related to the climate at test sites for either TempPC (*p* = 0.48) or PrecPC (*p* = 0.58).

### 3.2 Within‐population variation is negatively related to temperatures at trial site and population origin

The variation within populations CV_wp_ ranged from 0.12 to 0.58 for individual population–site combinations with an average of 0.28. Across single trial sites, the average CV_wp_ ranged from 0.14 to 0.41 (Table S1).

**Figure 5:**
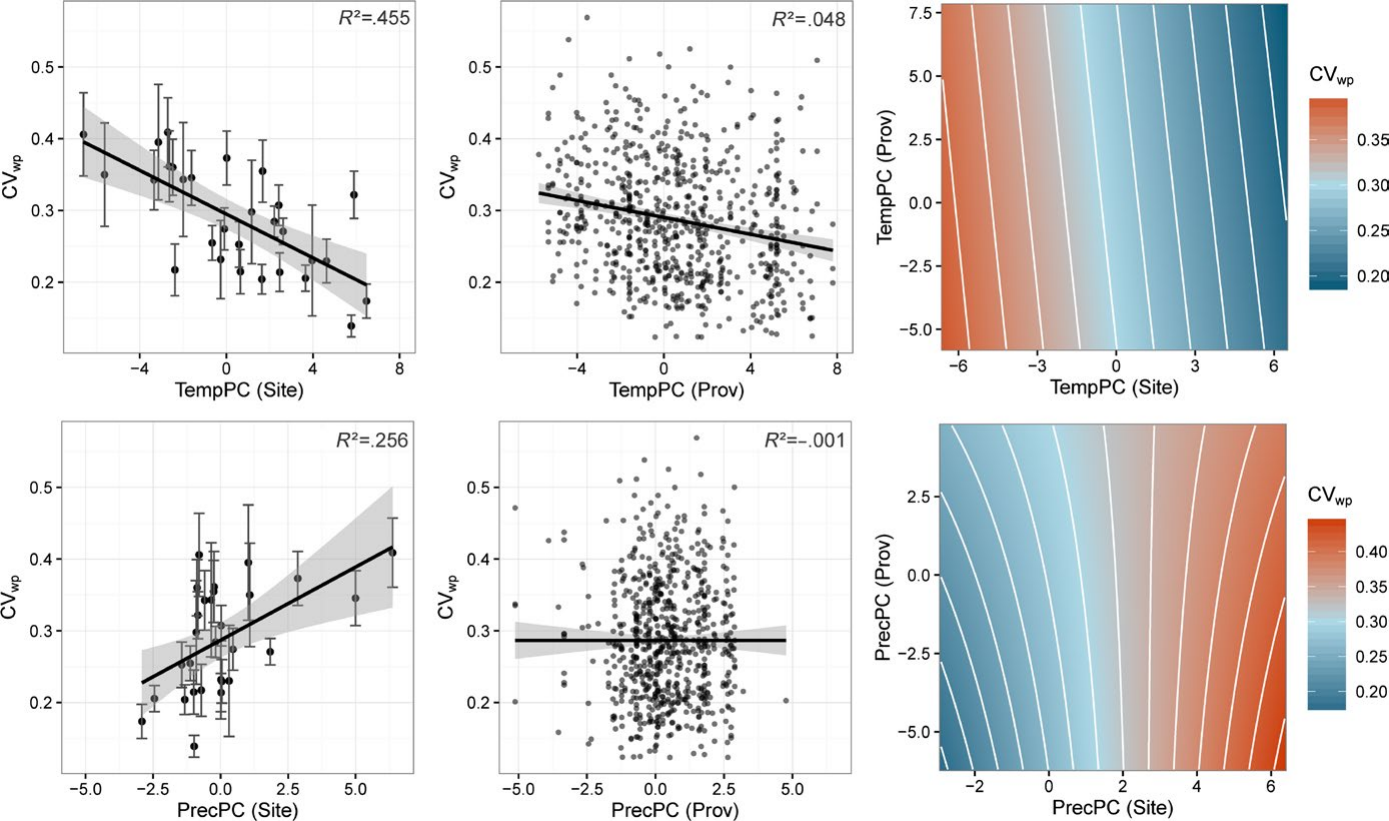
Within‐population variation for each population–site combination. Coefficients of variation for within‐population variation (CV_wp_) are shown along the climate gradients TempPC (top) and PrecPC (bottom), where large values of TempPC and PrecPC represent warm and wet locations, respectively. Left: response along climate at the test sites. Error bars represent CV_wp_ values from all populations tested at a respective site; Center: response along population climate. Points represent CV_wp_ values of one population tested at one specific site; Right: bivariate plot along both site and population climates. Color indicates the level of within‐population variation (CV_wp_) (see legend)

Multiple regression analysis revealed a significant relationship between the within‐population variation (CV_wp_) and the climate at the test sites and the population origin (see summary of the multiple linear model fit shown in Table 1). Regression analyses were performed separately for TempPC and PrecPC and for site and population‐origin climate (Fig. 5). Regressions of CV_wp_ to the site climate revealed negative relationships with TempPC and positive relationships with PrecPC. Therefore, at warm and dry sites, within‐population variation is low. In contrast, at cold and moist sites, within‐population variation is high (Fig. 5, left). Moreover, within‐population variation (CV_wp_) is negatively related to the climate of the population origin for TempPC (Table 1; Fig. 5, center).

**Table 1:** Parameter estimates of multiple regression analyses, predicting CV_wp_ by climate parameters TempPC and PrecPC at test sites (*_S) and population origins (*_P) (adjusted *R*² = 0.424, *F*‐statistic = 151.3 on 4 and 814 degrees of freedom)

	Estimate	*SE*	*t* value	Pr (>|*t*|)	
(Intercept)	0.2896	0.0024	123.1	<0.001	***
TempPC_S	−0.0116	0.0007	−15.5	<0.001	***
TempPC_P	−0.0017	0.0008	−2.1	0.0333	*
PrecPC_S	0.0134	0.0013	10.6	<0.001	***
PrecPC_P	0.0024	0.0014	1.8	0.0746	.

**Figure 6:**
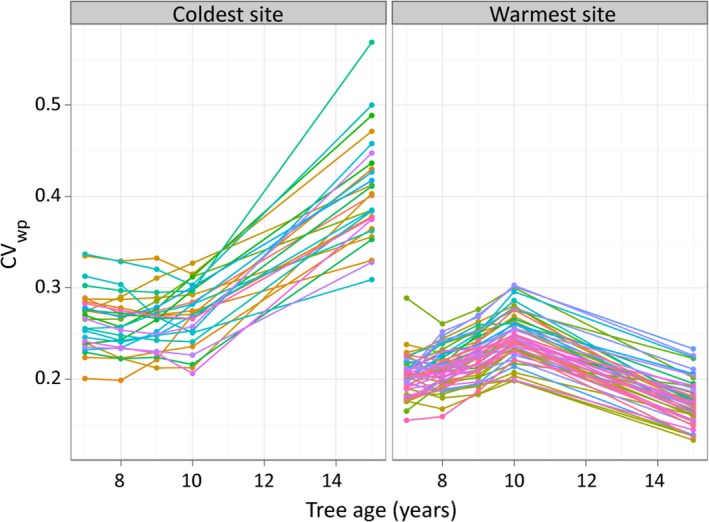
Within‐population variation and tree age. CV_wp_ of populations at climatically most extreme sites 11 (coldest) and 42 (warmest) at tree ages of 7, 8, 9, 10, and 15 years

The multiple regression analysis for the 15‐year‐old trees demonstrated clear effects of trial site climate on CV_wp_ after the trees had been growing for 10 years in the field. When the trees were planted at the age of 5 years, they had a similar variation within populations CV_wp_ at all test sites. To reveal the temporal course of CV_wp_ development throughout the growing period in the field, we also analyzed CV_wp_ for trees aged 7, 8, 9, and 10 years and compared CV_wp_ for populations at the coldest trial site (i.e., the lowest TempPC at site 11) and the warmest site (i.e., the highest TempPC at site 42) (Fig. 6). In 7‐year‐old trees, the variation within populations CV_wp_ after two growing periods was already higher at the coldest trial site than at the warmest site. At the coldest site, the within‐population variation increased from age of 10 to 15. A contrasting pattern was found at the warmest trial site on the upper temperature limit. Here, within‐population variation increased only slightly from tree ages of 7–10, but then remained constant at a relatively low level until the tree age of 15 years (Fig. 6).

**Figure 7:**
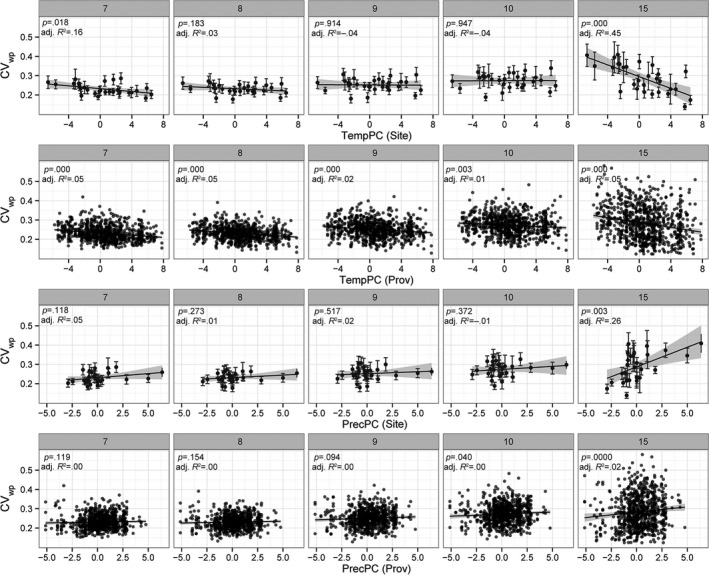
Within‐population variation, tree age, and climatic gradients. Values of CV_wp_ are shown along climatic gradients of TempPC (1st and 2nd row) and PrecPC (rows 3 and 4) for tree ages of 7, 8, 9, 10, and 15 years. Error bars in site climate responses (rows 1 and 3) represent within‐population variations of all populations on a site (mean ± *SD* of CV_wp_). Dots in population climate responses (rows 2 and 4) represent within‐population variation of a specific population at a specific site

These differences in CV_wp_ temporal development between trials at cold and warm sites can be observed at all trial sites (Fig. 7; the results of multiple regression analyses in Table S3): During the period from tree age of 7 to 15 years, CV_wp_ stayed at a more or less constant level at sites with warm (high TempPC) and dry (low PrecPC) climates, while CV_wp_ increased with tree age at sites with cold (low TempPC) and moist (high PrecPC) climates (Fig. 7, row 1, row 3). Thus, the significant relationships found between trial climate and CV_wp_ when a tree is 15 years old are due to increasing CV_wp_ at cold sites, but not to decreasing CV_wp_ at warm sites. We found the most pronounced response of CV_wp_ to site climate in 15‐year‐old trees. However, these relationships (including a significant linear regression) between sites climate and CV_wp_ were already found for trees at the age of 7, 8, 9, and 10 (Table S3), but the slope of the relationship increased with tree age.

Beyond a significant relation of CV_wp_ to site climate, we found a significant relation of CV_wp_ to the temperature‐related climate predictor (TempPC) of population origins for trees at the age of 7, 8, 9, 10, and 15 (Fig. 7, row 2; Table S3).

### 3.3 Shapes of density distributions of tree height

**Figure 8:**
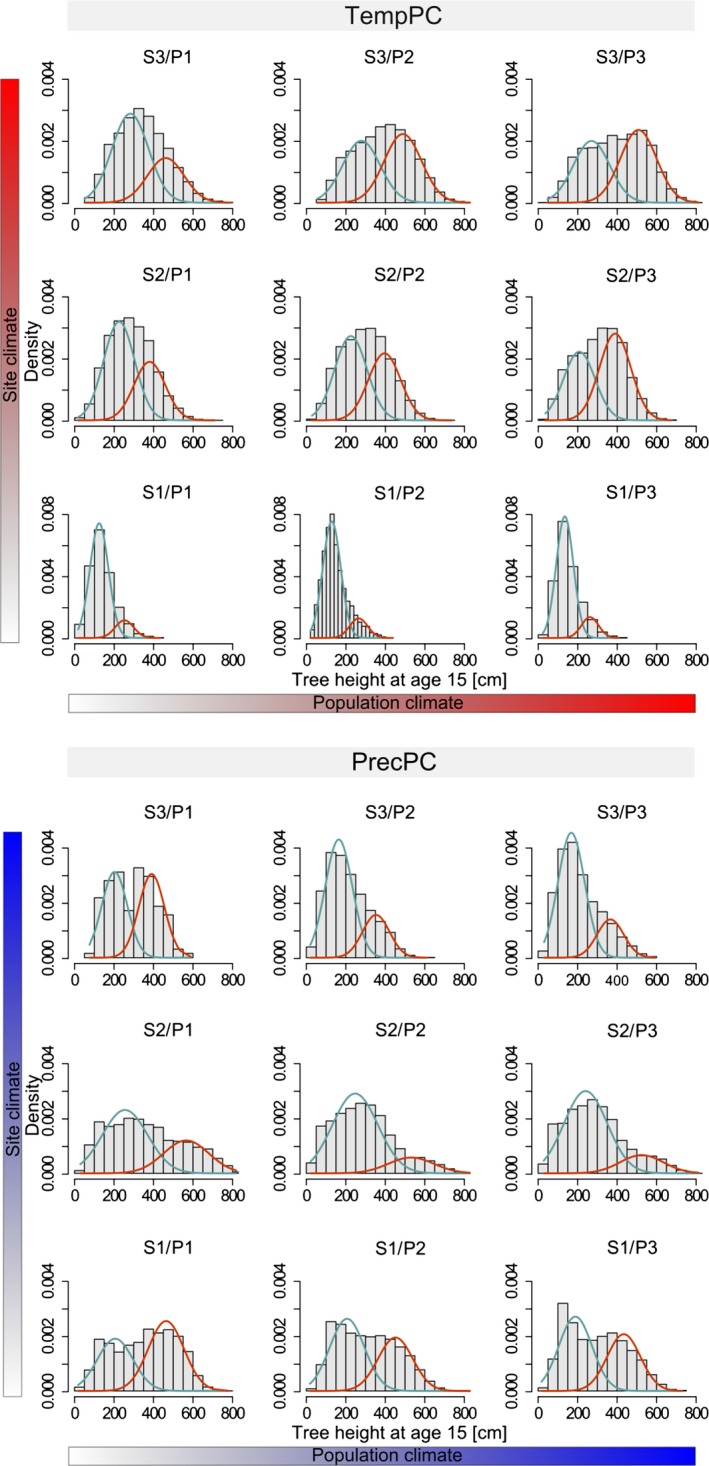
Density distributions of absolute tree heights at the age of 15. All records were divided into nine climate subsets by TempPC (top) and PrecPC (bottom). Each subset aggregates records of one of three test site groups (S1, S3, or S3) and one of three population groups (P1, P2, or P3). Red and blue curves are the results from a mixture‐model analysis and represent density probabilities of two hypothetical subcomponents for “small” and “high” trees within each subset. Parameter estimates from the mixture‐model analysis (portions of attributed data to each component, means of components and standard deviations) are presented in supplementary Table S4

The frequency density distributions of tree heights at the age of 15 reveal contrasting patterns for the categorized climatic subsets of the site and population climate. Figure 8 displays histograms of different site and population subsets (S1, S2, S3, P1, P2, P3) where the effects of the site climate are shown on the vertical axis and the effects of the climate at population's origins on the horizontal axis. With respect to site climate, we found increasing mean tree heights with increasing TempPC and decreasing PrecPC. The shape of the density distributions suggests that at warm and dry sites, the variation in tree heights is also larger than at cold and moist sites (in contrast to our previous analyses, where we used the coefficient of variation). We found tree height distributions at cold and moist sites to be left‐skewed, whereas at warm and dry sites they were not skewed or slightly right‐skewed (see Table 2 for mean, variance, skewness, and kurtosis of the distributions). Kurtosis refers to the presence or absence of pronounced peaks in a density distribution. At cold sites, we found “peaked” (leptokurtic) distribution shapes (positive kurtosis) and “flat” (platykurtic) distribution shapes (negative kurtosis) at warm and dry sites.

**Table 2:** Statistics describing forms of distribution of each of the nine combinations of climate subset (see Fig. 8)

S	P	Trees	Sites	Pop	Mean	Var	Skew	Kurt
*TempPC*								
1	1	8,019	8	60	141.4	4,084.1	0.87	0.92
1	2	9191	8	82	147.7	4,356.0	0.96	0.93
1	3	2346	8	20	153.1	3,929.0	0.95	0.94
2	1	7457	11	45	282.8	11,796.4	0.25	−0.39
2	2	15436	11	109	300.4	13,894.7	0.17	−0.57
2	3	4811	11	29	309.1	14,496.8	0.00	−0.69
3	1	6109	10	35	341.8	15,792.7	0.30	−0.35
3	2	21266	10	122	388.8	20,029.1	0.03	−0.70
3	3	9413	10	38	398.4	22,514.9	−0.04	−0.87
*PrecPC*								
1	1	3,635	18	17	353.5	24,419.5	−0.10	−1.04
1	2	39,289	21	184	310.5	22,335.8	0.28	−0.89
1	3	15,619	21	74	294.8	21,815.6	0.31	−0.91
2	1	1,225	4	9	361.3	34,765.4	0.35	−0.81
2	2	11,230	6	90	293.9	24,381.4	0.60	−0.02
2	3	7,081	6	74	291.8	23,994.6	0.62	−0.07
3	1	129	1	1	295.4	13,147.1	0.15	−0.91
3	2	3,436	2	29	214.4	11,627.4	0.60	−0.28
3	3	2,404	2	19	214.3	11,596.1	0.75	−0.01

S, P = IDs of the climate subsets of sites and populations indicating the levels of the respective climate variable TempPC or PrecPC (1 = low, 2 = medium, 3 = high); trees = the number of individual trees pooled in the combination of climate subsets; sites = the number of pooled sites; pop = the number of pooled populations; mean = mean tree height at the age of 15; var = variance of tree heights; skew = skewness of tree height distribution; kurt = kurtosis of tree height distribution

Population subsets (P1, P2, P3) reveal only small differences in histogram patterns (Fig. 8). Overall, the mean tree height and the variation increased from (cold) P1 populations to (warm) P3 populations. Moreover, skewness increases from cold/moist populations to warm/dry populations, whereas kurtosis decreases. Thus, the populations from cold/moist sites are rather left‐skewed and peaked, whereas the populations from warm/dry sites are nonskewed or slightly right‐skewed and flat.

Density probability functions from mixture analyses provide additional insights into these patterns. In mixture analysis, each tree in a subset combination is attributed to one of two hypothetical components of the distribution (curves in Fig. 8), representing “relatively small” and “relatively tall” trees. Means and standard deviations of each component, as well as probabilities of data being classified as one of these components, are shown in Table S4. At warm S3 sites (high TempPC), 67% of trees from cold populations are classified as small (blue curve), while 33% of trees are classified as tall (red curve). Trees from warm populations at the same S3 sites are classified as 46% small and 54% tall. At intermediate S2 sites, we again have a larger number of “small trees” in P1 populations, but conversely a larger number of “tall trees” in P3 populations. At cold S1 sites, most trees are classified as small, regardless of population climate.

### 3.4 Robustness to stratification

To avoid the biases in our analyses due to the unbalanced distribution of populations to trial sites, we corrected our dataset by stratifying population x site combinations according to their relative frequency in the bivariate spectrum of the population and site climate (see Fig. 3). The reanalysis of the multiple linear regression analyses of the response variable CV_wp_ and the predictors TempPC and PrecPC of site and population origins (Table 1) with the two stratification weights (weight#1 and weight#2) did not alter the significance of the regression analysis compared to the unweighted model (Table S5). Using weight#1 resulted in a higher significance level for TempPC of population origin, and using weight#2 resulted in a higher significance level even for PrecPC of population origin. Therefore, stratification by using two different weighting methods did not reveal any false significant model parameters in the linear regression analysis of the unweighted data. Instead, both weighted models resulted in a slightly stronger relevance of TempPC of population origins (compare Table 1 and Table S5).

### 3.5 Effect of mortality

The analysis of survival rates across our trial sites did not indicate a bias of tree height variation measures. A significant relationship was found between the survival rate (calculated as the proportion of surviving trees after 10 years in the field) and TempPC of the trial sites, but not to TempPC of provenance origin. However, the total effect of site climate on mortality seems to be negligible, as the coefficient of determination of this relationship is very low (adjusted R² = 0.023) and the regression slopes close to zero (Table 3). Also, the relation of survival to PrecPC was not found to be significant for trial sites and provenance origin.

**Table 3:** Parameter estimates of multiple regression analyses predicting the survival rate by climate parameters TempPC and PrecPC at test sites (*_S) and population origins (*_P)

	Estimate	*SE*	*t* value	Pr (>|*t*|)	
(Intercept)	0.9713	0.0093	104.03	<0.001	***
TempPC_S	0.0131	0.0029	4.41	<0.001	***
TempPC_P	−0.0058	0.0032	−1.78	0.0757	.
PrecPC_S	0.0125	0.0050	2.48	0.0135	*
PrecPC_P	0.0090	0.0054	1.69	0.0923	.

Survival rate was calculated for each population–site combination as the percentage of living trees after 10 years in the field. We used arcsine transformation to meet normality assumptions. Adjusted *R*² = 0.023, *F*‐statistic = 5.73 on 4 and 814 *df*.

## 4 Discussion

The persistence of tree populations in future climates depends crucially on their adaptive capacity to adjust to new environmental conditions. Phenotypic variation in fitness‐related traits is both a result of environmental selection pressures and a prerequisite for the adaptation to changing environments. Here, we used one of the largest common garden trial series of Norway spruce established more or less throughout its complete climate distribution in Central Europe and tested for the effects of climate conditions on the phenotypic variation within and among populations. Both the climate at trial sites and the climate at the geographic origin of populations had a significant impact on the phenotypic variation within populations (Fig. 5). The effect of site climate on phenotypic variation was much larger than the effect of climate of population origins, as previously shown also for other conifers (Chakraborty et al., 2015; Wang et al., 2010). The observed effect of site climate suggests stronger climatic selection pressures at the colder end of the species distribution resulting in increasing tree height variation in colder and moister environments. Survival rates were neither affected by site climate nor by provenance origin climate (see the numbers of planted and measured trees in Table S1).

### 4.1 Environmental sensitivity of selection

Absolute mean heights were found to be strongly associated with site climate as a result of phenotypic plasticity. At the age of 15, trees reached on average 5 m at the warmest site and 1.2 m at the coldest site. Trees at warm sites also showed a higher absolute variance of tree heights. However, the overall coefficient of variation per trial site decreased significantly with increasing temperatures and ranged from 0.49 at the coldest site to 0.15 at the warmest (Fig. 4). This higher variation at colder sites was found to result mainly from the variation within populations (CV_wp_), because the ratio of variation among populations to the total variation was not related to the site climate.

The strongest relationship between CV_wp_ and climate was found at tree age of 15 years (i.e., growing for 10 years in the field). The analysis of the temporal course of CV_wp_ by including data from earlier height measurements showed that CV_wp_ increased from age 7 to age 15 at the coldest trial sites, whereas CV_wp_ remained approximately constant at trial sites at the warm end of the species distribution (Figs. 6 and  7). This suggests that the climate conditions at the colder trial sites increase the differentiation among trees within populations, but climate conditions at warm sites do not affect CV_wp_. Thus, at cold sites, relatively few well‐performing trees have an advantage in height growth and will likely dominate and outcompete the slower‐growing trees in the future stand. Given the huge intraspecific competition among juvenile trees (Vieilledent, Courbaud, Kunstler, & Dhôte, 2010), this will ultimately result in a higher potential selection differential at the cold edge of the spruce distribution. This is also supported by the mixture‐model analysis across different environments (Fig. 8 and Table S4). At cold sites (labeled S1 in Fig. 8), we found right‐skewed, leptokurtic distributions with a pronounced peak (negative kurtosis) resulting in a prevailing component of relatively small trees and a second much smaller component of taller trees. If climate was of similar importance at the upper temperature limit of the species' niche, we would expect a left‐skewed distribution at the warmer trial sites (labeled S3 in Fig. 8), where physiological limits set a threshold for height growth. Instead, we did not find left‐skewed distributions at the warmest trial sites, but nonskewed, platykurtic (positive kurtosis) distributions without pronounced peaks (Fig. 8, top). From such shapes of tree height density distributions, we conclude that Norway spruce is not under strong selection by climate constraints at any of our warm‐dry test sites—some of which are located at the upper temperature limit of the species range (Fig. 1).

Potential reasons for the increasing height variation within populations at cold sites are manifold. One possible explanation for the increasing height variation at colder sites could be the strong relation between height growth and phenology (e.g., Kleinschmit, Sauer‐Stegmann, Lunderstadt, & Svolba, 1981). In particular, tree height is strongly correlated with flushing and bud set within and across populations. Both traits, flushing and bud set, possess high additive genetic variation (Hannerz et al., 1999) and are driven by the annual temperature course in spring and late summer (Søgaard, Oystein, Jarle, & Olavi, 2008). Thus, at cold sites, small differences in temperature sum requirements for flushing among individual trees may accumulate into large differences in the onset of growth and resulting height growth pattern. As temperature accumulates much faster at warm sites, the same genetic differences in temperature sum requirements result in smaller differences of bud burst and height growth in warmer environments. Such behavior was also observed for other species: Davi et al. (2011), for example, analyzed flushing of various tree species from 960 to 1530 m a.s.l. and found a significant altitude effect for *Pinus sylvestris,* which decreased with faster spring development. Generally, our results on climate constraints at the cold end of the species distribution are in good agreement with the manifold traits that were found to be related to cold adaptation in conifers (Aitken & Hannerz, 2001; Howe et al., 2003; Morgenstern, 1996). Besides bud burst and bud set, a significant variation within and among families of Norway spruce has been found for frost hardiness (Skrøppa, 1991) and populations from southern Finland were found to be more sensitive to frost events than northern populations (Pulkkinen, 1993). Narrow‐sense heritability of frost resistance ranged from 0.04 to 0.28 in a Swedish progeny trials (Hannerz et al., 1999). Howe et al. (2003) concluded that cold adaptation traits appear to be under strong natural selection. In contrast, evidence for genetic adaptations of Norway spruce to warm temperatures, namely drought resistance or stomatal conductance, is limited. In part, this may be due to the fact that fewer studies have addressed the intraspecific variation in adaptation to the warm temperature edge of a species' range (but see Mátyás, Nagy, & Jármay, 2009 or Lamy et al., 2011); but it may also be that Norway spruce rarely reaches its physiological limits and has thus developed fewer local adaptations. Another potential cause for the unequal climate selection could be the structure of the trial sites itself: Warm trials are rather located at low elevations with relatively flat and homogeneous site conditions. In contrast, cold trial sites are located on mountain slopes and likely provide higher on‐site heterogeneity that may result into increasing differentiation among individual trees. A larger heterogeneity of both land surface and soil structure might provide advantageous microclimatic or edaphic conditions under which a few young trees are able to outperform their competitors. Also, snow cover in early spring is likely to increase the variation among trees, as it may delay the onset of growth for small trees completely covered with snow in particular. Larger trees that extend already beyond the snow cover are able to receive environmental signals for the start of the growing period much earlier than their smaller counterparts.

### 4.2 Phenotypic evolution and environmental heterogeneity

Besides the climate at the trial sites, the climate of population origin was also related to CV_wp_ with higher variations within populations from colder regions (Fig. 7, row 2; Table S3). This finding seems counterintuitive, considering that with stronger selection at colder sites one might expect lower phenotypic variation. One explanation could be the ongoing phenotypic evolution at a marginal habitat and within changing environments where, for large and recombining populations, the genetic variance was found to increase under directional selection as a result of an increasing frequency of rare alleles (e.g., Burger, 1999; Burger & Lynch, 1995). This type of situation has been observed in several theoretical studies and may fit for Norway spruce at its cold edge, namely at higher elevations in its alpine distribution. Here, permanent selection pressures might cause maladaptations and thus a permanent lag of the population mean behind the environmental optimum (see Kopp & Matuszewski, 2014). Another explanation for the increased variation could be the immediate effect of phenotypic plasticity, which was found to increase the genetic variation within one generation because of variations in the slope of reaction norms (Chevin & Lande, 2011). To differentiate between the effects of phenotypic plasticity or directional selection as described by Burger (1999), the immediate effects of mortality during changes in the genetic variance need to be considered. Our analyses of survival rates at trial sites indicated only a negligible effect of climate on mortality, as the coefficient of determination of this analysis is very low (adjusted R² = 0.023) and regression slopes are close to zero (Table 3). This indicates that between the age of 5–15 years, tree height variation is rather shaped by phenotypic plasticity than natural selection. Theoretical analysis of plasticity and phenotypic variation in populations within marginal habitats along environmental gradients support this conclusion. Chevin and Lande (2011), for example, found an increase in genetic variance within one generation. However, the effects of changing environments on variance and the contributions of plasticity are still not fully understood, because genetic variances are often assumed to be constant in quantitative models (Kopp & Matuszewski, 2014). Another explanation for higher variation within populations from colder origins could be intensive gene flow and environmental heterogeneity in such environments. In our experiment, cold populations originate mainly from alpine locations at higher elevations. Within its distribution in the Alpine region, Norway spruce occurs from the valley floors up to subalpine habitats near the tree line. This high environmental heterogeneity likely results in manifold local adaptations. The intense gene flow caused by pollen flow across spatially close but climatically distant populations could increase the genetic variation by introducing maladapted genotypes, as shown, for example, by Yeaman and Jarvis (2006). The gene flow and environmental heterogeneity explanation is supported by a recent meta‐analysis of progeny tests of Norway spruce in Sweden (Kroon, Ericsson, Jansson, & Andersson, 2011). This study compared the genetic variation across a latitudinal gradient from 56°N to 65°N covering a similar climatic range. In contrast to our study, Kroon et al. (2011) found a significant decrease in genetic variation with increasing latitude and thus decreasing temperatures. Across the large spatial distance in Sweden, gene flow is much less likely to connect the same environmental gradients as in the Alpine landscape.

### 4.3 Among‐population variation

The ratio of among‐population variation to the total phenotypic variation ranged from 25% to 73% (with a mean of 47%, Table S1) and did not change along the climatic gradient of TempPC or PrecPC. This is in agreement with our initial hypothesis that the distribution of populations across trial locations (although not completely balanced) results in similar differences between populations at each site. However, the higher selection differential acting on within‐population variation at colder trial sites would imply that selection also increases the differences among populations. Instead, the ratio of among‐population variation was found to be independent from site climate, indicating that besides local adaptations to climate, other factors might contribute to differentiation among populations. Such a factor is most likely the phylogeographic pattern of *P. abies*. The majority of tested populations originated from the eastern Alpine range and from surrounding countries. Indeed, the geographic origins of the populations cover the three main refugial lineages of Norway spruce in Central and Western Europe. The long population history of these lineages can still be observed with various molecular markers (e.g., Maghuly, Pinsker, Praznik, & Fluch, 2006; Mengl, Geburek, & Schueler, 2009), and provenance trials throughout Europe have recognized a strong variation among regional groups (Giertych, 1992; Krutzsch, 1992). In comparison with other provenance experiments with Norway spruce, the provenance effects on tree height variation in our study (25%–73%) seems higher than observed elsewhere (e.g., Weisgerber, Dimpflmeier, Ruetz, Kleinschmitt, & Widmaier, 1984; : 44%; Liesebach, Rau, & König, 2010: 0%–26%; Ujvári‐Jármay, Nagy, & Mátyás, 2016: 12.6%). This is, however, because we calculated the coefficient of variation in order to correct for the strong variation in height growth among trial sites. The untransformed ratio of the among‐population variation to total phenotypic variation ranged from 6% to 53% and is comparable to the values reported by Weisgerber et al. (1984), Liesebach et al. (2010), and Ujvári‐Jármay et al. (2016).

### 4.4 Understanding Norway spruce range limits

The focus of our empirical study is on tree height data, but not on direct measures of a tree's fitness in terms of survival and reproduction. Such direct measures of fitness for tree species are difficult to obtain, because many forest trees do not flower below the age of 20 years and unbiased estimates of reproductive performance would require reproductive success to be measured throughout an individual tree's life cycle. Nevertheless, measures of growth performance at the juvenile age are considered to be closely connected to fitness, as the intraspecific competition among trees is strongest among seedlings and juvenile trees (Vieilledent et al., 2010); only the very few trees that are able to dominate others will win the race for light and survive. During periods of extreme environmental conditions (e.g., drought or frost) as well as at later life stages, other physiological or phenological traits may have a higher impact and obscure height growth performance, but then the selection for tree height is expected to have already shaped the genetic structure of populations.

Under the assumption that tree height is strongly correlated with fitness, the varying selection differential at opposite ends of the spruce distribution can be discussed in terms of the species' fundamental and realized niche. At the cold end of the species distribution, we see high selection differential that very likely translates into a sharp range margin. Polechova and Barton (2015) recently demonstrated such an intrinsic limit to adaptation by modeling the joint evolution of trait mean and population size along environmental gradients within a genetic model. The existence of such range margins depends on the relation between fitness costs and the efficacy of selection relative to genetic drift (Polechova & Barton, 2015). Even small environmental gradients are able to generate intrinsic genetically determined range limits in the case of interspecific competition (Case & Taper, 2000), which tends to be the reality in a subalpine forest ecosystem. Our data neither allow us to estimate the model parameters of Polechova and Barton (2015) nor to prove the effects of interactions with other conifers, but they clearly demonstrate that climate selection in Norway spruce acts at the cold, but not the warm species distribution limit. Thus, the colder species distribution limit can be considered close to the limits of its fundamental niche, whereas the warm limit rather mirrors the species realized niche. Here, the species distribution might instead be defined by drivers other than climate, such as bark beetle attacks or competition with other tree species, which were found to be important drivers of species niches on ecological timescales (Hellmann, Prior, & Pelini, 2012; Meier, Edwards, Kienast, Dobbertin, & Zimmermann, 2011). However, given the short observation time in relation to a trees life span, it is also possible that we are not able to identify direct causes of mortality, or reduced reproduction at the warm temperature limit, which might also be related to intrinsic limits of the species range. To test whether the observed differences in selection differential are being carried into adult tree populations, we could design a simple genetic experiment: as our analysis predicts that no selection on tree height occurs on populations growing on the warm temperature limit, we would expect low heritability for height growth performance in the offspring of such trees. In contrast, offspring from populations growing at cold sites should have a higher degree of genetic determination.

Under climate change, the cold distribution limit is the leading edge that might spread to higher altitudes and latitudes (Sykes et al., 1996). The warm distribution limit is considered to be the trailing edge, where Norway spruce is expected to experience strong reductions in its present range with significant consequences for forest ecosystems, wood production (Hanewinkel et al., 2012), but also with losses of genetic diversity (Schueler et al., 2014). As our analysis indicates that natural selection at this trailing edge is limited, local adaptation at such sites seems to be impossible, in particular if we consider the high velocity of change. Thus, management actions to conserve the existing genetic diversity, for example, the establishment of gene conservation forests, are urgently needed at the warm limit of the species distribution range.

### 4.5 Implications for forest tree breeding and distribution modeling

Our analysis aims to improve tree genetic conservation and to guide assisted migration measures. Assisted migration, that is, the translocation of forest reproductive material to areas with expectably favorable climates in the future, is widely discussed as a key forest management strategy to reduce climate maladaptation (e.g., Lu et al., 2014; McLachlan, Hellmann, & Schwartz, 2007; Wang et al., 2010). Based on the same Norway spruce dataset, we have already shown that populations from currently warm and drought‐prone areas are appropriate candidates for continued silvicultural use in the future (Kapeller et al., 2012). In the mountainous area of the eastern Alps, this mainly means a shift of seed material upward in order to keep pace with global warming. In the present analysis, we found significantly stronger selection differential at the species colder distribution limit, although this has not resulted in reduced genetic variation in populations originating from such sites, likely because of intensive gene flow and environmental heterogeneity in alpine environments. This suggests that reasonable seed transfers upward bears only a small risk of maladaptations, as the variation within the populations is only weakly correlated with the temperature gradient, and thus, also populations from warmer seed origin display a broad adaptive capacity to grow and survive on colder sites. In general, however, genetic conservation and seed transfer activities should not be based on the variation in single traits alone. A valid risk‐benefit analysis might also consider further physiological or phenological traits related to the adaptive potential of populations (e.g., frost hardiness, drought resistance, and pest insect tolerance).

Similarly, attempts to model future tree species distributions need to take into account the variable selection differential along climatic gradients and the relative importance of adaptive traits at specific areas within the species range. For Norway spruce, it is widely believed and shown with various species distribution models that the species will undergo strong reductions in its present range mainly on the species' warm and dry distribution limit (e.g., Hanewinkel et al., 2012; Sykes et al., 1996; Zimmermann et al., 2013). This is in contrast to our data as they do not show signs of climate selection on tree height on warm trial sites, whereas cold conditions impose a stronger selection with highly skewed density functions. Thus, our analysis helps to decipher individual mechanisms that may trigger range contractions or expansions that should be included in future mechanistic distribution models. This will help to improve models of the species' fundamental niche because under climate change it is more important to understand where a species *could* occur than where it *currently* occurs (Wiens, Stralberg, Jongsomjit, Howell, & Snyder, 2009).

## 5 Conclusions

Under climate change, populations throughout the entire climatic range will experience shifts of mean temperatures, related climate parameters, or both. In Norway spruce, populations at the currently cold sites harbor higher phenotypic variation and will likely be able to adapt to the prospective conditions. At the warm edge of its distribution, populations are not necessarily maladapted, as we have not observed climatic constraints on phenotypic variation even in our warmest trial sites at the border of the climatic range. In our analysis, temperature shapes the phenotypic variation much more strongly than precipitation‐related parameters. As climatic predictions for temperature are more reliable than for precipitation, our results could be integrated into mechanistic models of population persistence and species distributions. Datasets similar to those used in the present study are available for many tree species and have been used to select appropriate populations for future reforestations based on the population's mean climate response (e.g., Leites, Robinson, Rehfeldt, Marshall, & Crookston, 2012; Lu et al., 2014; Rehfeldt, Tchebakova, & Barnhardt, 1999; Rehfeldt et al., 2001, 2002). Our analysis suggests that an in‐depth analysis of the phenotypic variation within such datasets can provide additional knowledge on the population's adaptive capacities.

## Data Arching Statement

Data files used for the analyses in this study are available at the Dryad Digital Repository: doi: http://dx.doi.org/10.5061/dryad.877ts.
