# Supplementary material for: Varying selection differential throughout the climatic range of Norway spruce in Central Europe
Source: Evol Appl. 2016 Oct 23;10(1):25–38. doi: 10.1111/eva.12413 (PMC5192884; doi:10.1111/eva.12413)
Supplement: Supplementary file 1 [file EVA-10-25-s001.docx]

Table S1. Overview of site specifications and variance components for each trial site. Site= ID of respective site; TempPC = temperature related principal component; PrecPC = precipitation related principal component; Pops = number of tested populations; Blocks = number of repetitions; Trees_0 = number of trees planted at trial start;Trees_15 = number of trees measured at age 15; µ_s_= mean tree height at age 15; σ²_s_ = total variance of tree heights at age 15; σ²_ap_ = variance component explained by populations; σ²_b_ = variance component explained by blocks;σ²_res_ = residual variance component;CV_s_,CV_ap_, CV_b_, CV_res_= standardized variancecomponents as coefficients of variation; CV_ap_/CV_S,_ CV_b_/CV_s_, CV_res_/CV_s_ = ratios of standardized variancecomponents to total standardized variance; MCVwp = mean within-population variation (of all populations per site)

| Site | TempPC | PrecPC | Pops | Blocks | Plots | Trees_0 | Trees_15 | µ_s_ | σ²_s_ | σ²_ap_ | σ²_b_ | σ²_res_ | σ²_p_/σ²_s_ |  | CV_s_ | CV_ap_ | CV_b_ | CV_res_ | CV_ap_/CV_s_ | CV_b_/CV_s_ | CV_res_/CV_s_ | MCV_wp_ |
| --- | --- | --- | --- | --- | --- | --- | --- | --- | --- | --- | --- | --- | --- | --- | --- | --- | --- | --- | --- | --- | --- | --- |
| 1 | -2,0 | -0,3 | 31 | 1 | 31 | 1501 | 1237 | 194 | 9055 | 4803 | 0 | 4252 | 0,53 |  | 0,49 | 0,36 | 0,00 | 0,34 | 0,73 | 0,00 | 0,69 | 0,34 |
| 7 | -3,1 | 1,0 | 26 | 3 | 78 | 3159 | 1960 | 147 | 4587 | 1892 | 302 | 2393 | 0,41 |  | 0,46 | 0,30 | 0,12 | 0,33 | 0,64 | 0,26 | 0,72 | 0,40 |
| 8 | -5,6 | 1,1 | 24 | 3 | 72 | 3329 | 1443 | 80 | 1015 | 292 | 70 | 652 | 0,29 |  | 0,40 | 0,21 | 0,10 | 0,32 | 0,54 | 0,26 | 0,80 | 0,35 |
| 9 | -0,7 | -1,1 | 23 | 3 | 69 | 2827 | 1974 | 330 | 7992 | 1128 | 174 | 6690 | 0,14 |  | 0,27 | 0,10 | 0,04 | 0,25 | 0,38 | 0,15 | 0,91 | 0,25 |
| 10 | -2,5 | -0,9 | 26 | 3 | 78 | 3650 | 3169 | 202 | 6024 | 1226 | 248 | 4550 | 0,20 |  | 0,38 | 0,17 | 0,08 | 0,33 | 0,45 | 0,20 | 0,87 | 0,36 |
| 11 | -6,6 | -0,8 | 27 | 3 | 81 | 2630 | 2099 | 116 | 2571 | 482 | 156 | 1932 | 0,19 |  | 0,44 | 0,19 | 0,11 | 0,38 | 0,43 | 0,25 | 0,87 | 0,41 |
| 12 | 1,2 | -0,9 | 23 | 3 | 69 | 3586 | 1516 | 355 | 13294 | 4404 | 844 | 8046 | 0,33 |  | 0,33 | 0,19 | 0,08 | 0,25 | 0,58 | 0,25 | 0,78 | 0,30 |
| 14 | 0,6 | -1,0 | 27 | 3 | 81 | 3731 | 2937 | 424 | 9703 | 2112 | 418 | 7173 | 0,22 |  | 0,23 | 0,11 | 0,05 | 0,20 | 0,47 | 0,21 | 0,86 | 0,21 |
| 15 | -0,1 | 0,5 | 26 | 3 | 78 | 3535 | 3222 | 284 | 6768 | 1299 | 77 | 5391 | 0,19 |  | 0,29 | 0,13 | 0,03 | 0,26 | 0,44 | 0,11 | 0,89 | 0,27 |
| 20 | 0,0 | 2,9 | 26 | 1 | 26 | 3356 | 2836 | 195 | 5695 | 355 | 0 | 5340 | 0,06 |  | 0,39 | 0,10 | 0,00 | 0,37 | 0,25 | 0,00 | 0,97 | 0,37 |
| 23 | -3,3 | -0,6 | 26 | 3 | 78 | 3000 | 2653 | 143 | 2634 | 389 | 105 | 2140 | 0,15 |  | 0,36 | 0,14 | 0,07 | 0,32 | 0,39 | 0,20 | 0,90 | 0,34 |
| 24 | -0,3 | 0,0 | 26 | 2 | 52 | 2425 | 1972 | 302 | 5895 | 1596 | 462 | 3837 | 0,27 |  | 0,25 | 0,13 | 0,07 | 0,21 | 0,52 | 0,28 | 0,81 | 0,23 |
| 25 | -2,4 | -0,7 | 49 | 3 | 147 | 4685 | 3146 | 131 | 989 | 188 | 12 | 790 | 0,19 |  | 0,24 | 0,11 | 0,03 | 0,21 | 0,44 | 0,11 | 0,90 | 0,22 |
| 26 | -2,6 | -0,2 | 25 | 3 | 75 | 2903 | 2330 | 170 | 4127 | 646 | 377 | 3104 | 0,16 |  | 0,38 | 0,15 | 0,11 | 0,33 | 0,40 | 0,30 | 0,87 | 0,36 |
| 27 | 2,2 | -0,2 | 25 | 3 | 75 | 3646 | 3030 | 187 | 3047 | 281 | 90 | 2677 | 0,09 |  | 0,30 | 0,09 | 0,05 | 0,28 | 0,30 | 0,17 | 0,94 | 0,28 |
| 29 | 1,7 | -0,3 | 24 | 3 | 72 | 3422 | 3022 | 186 | 4963 | 1387 | 59 | 3516 | 0,28 |  | 0,38 | 0,20 | 0,04 | 0,32 | 0,53 | 0,11 | 0,84 | 0,35 |
| 30 | 4,0 | 0,3 | 36 | 3 | 108 | 4069 | 3495 | 515 | 24705 | 10103 | 3930 | 10672 | 0,41 |  | 0,31 | 0,20 | 0,12 | 0,20 | 0,64 | 0,40 | 0,66 | 0,23 |
| 31 | -1,6 | 5,0 | 27 | 3 | 81 | 3735 | 3230 | 277 | 10055 | 2453 | 208 | 7394 | 0,24 |  | 0,36 | 0,18 | 0,05 | 0,31 | 0,49 | 0,14 | 0,86 | 0,35 |
| 32 | -2,7 | 6,4 | 24 | 3 | 72 | 3776 | 2643 | 140 | 3552 | 545 | 96 | 2911 | 0,15 |  | 0,43 | 0,17 | 0,07 | 0,39 | 0,39 | 0,16 | 0,90 | 0,41 |
| 33 | 2,4 | 0,0 | 25 | 3 | 75 | 3620 | 2985 | 240 | 5825 | 704 | 36 | 5086 | 0,12 |  | 0,32 | 0,11 | 0,02 | 0,30 | 0,35 | 0,08 | 0,93 | 0,31 |
| 34 | 2,5 | 0,0 | 33 | 3 | 99 | 5016 | 4699 | 412 | 9400 | 2251 | 850 | 6300 | 0,24 |  | 0,24 | 0,12 | 0,07 | 0,19 | 0,49 | 0,30 | 0,82 | 0,21 |
| 35 | 2,6 | 1,8 | 50 | 3 | 150 | 8008 | 6426 | 324 | 8233 | 795 | 78 | 7360 | 0,10 |  | 0,28 | 0,09 | 0,03 | 0,27 | 0,31 | 0,10 | 0,95 | 0,27 |
| 36 | 4,6 | 0,0 | 19 | 3 | 57 | 2995 | 2051 | 421 | 10810 | 2254 | 102 | 8454 | 0,21 |  | 0,25 | 0,11 | 0,02 | 0,22 | 0,46 | 0,10 | 0,88 | 0,23 |
| 39 | 1,6 | -1,3 | 22 | 3 | 66 | 3666 | 3043 | 385 | 7719 | 1652 | 242 | 5826 | 0,21 |  | 0,23 | 0,11 | 0,04 | 0,20 | 0,46 | 0,18 | 0,87 | 0,20 |
| 40 | 0,6 | -1,4 | 25 | 3 | 75 | 3679 | 2477 | 320 | 7845 | 1748 | 323 | 5774 | 0,22 |  | 0,28 | 0,13 | 0,06 | 0,24 | 0,47 | 0,20 | 0,86 | 0,25 |
| 41 | 3,7 | -2,5 | 24 | 3 | 72 | 3585 | 2771 | 370 | 6713 | 1278 | 51 | 5384 | 0,19 |  | 0,22 | 0,10 | 0,02 | 0,20 | 0,44 | 0,09 | 0,90 | 0,21 |
| 42 | 6,5 | -2,9 | 53 | 3 | 159 | 8658 | 7133 | 496 | 8486 | 1622 | 85 | 6779 | 0,19 |  | 0,19 | 0,08 | 0,02 | 0,17 | 0,44 | 0,10 | 0,89 | 0,17 |
| 43 | 5,8 | -1,0 | 22 | 3 | 66 | 3234 | 1512 | 519 | 5803 | 832 | 57 | 4914 | 0,14 |  | 0,15 | 0,06 | 0,01 | 0,14 | 0,38 | 0,10 | 0,92 | 0,14 |
| 44 | 5,9 | -0,8 | 25 | 3 | 75 | 3675 | 2293 | 267 | 9452 | 1740 | 1544 | 6168 | 0,18 |  | 0,37 | 0,16 | 0,15 | 0,29 | 0,43 | 0,40 | 0,81 | 0,32 |

**Table S2.** Contribution of climate variables to each dimension of the principal component analysis.Parameters included in this analysis were: longitude (lon), latitude (lat), altitude (alt), length of vegetation period (VP),average day of first frost in fall (FF), annual heat-moisture index (AHM, see Wang et al. 2006), growing degree days (GDD), mean winter temperature (WT), mean temperature (Tm), temperature maximum (Tx), temperature minimum (Tn) and mean precipitation sums (P); the latter parameters are each given during vegetation season (.Veg) and outside of vegetation season (.NVeg).

|  | ***TempPC*** | ***PrecPC*** | ***Dim3*** | ***Dim4*** | ***Dim5*** |
| --- | --- | --- | --- | --- | --- |
| lon | 0.265 | 0.495 | 0.436 | 0.589 | 0.365 |
| lat | 0.344 | 0.517 | 0.625 | 0.260 | 0.265 |
| alt | 0.941 | 0.131 | 0.225 | 0.083 | 0.104 |
| VP | 0.968 | 0.087 | 0.058 | 0.110 | 0.067 |
| FF | 0.923 | 0.237 | 0.005 | 0.148 | 0.063 |
| AHM | 0.615 | 0.730 | 0.173 | 0.069 | 0.044 |
| GDD | 0.653 | 0.017 | 0.164 | 0.445 | 0.557 |
| WT | 0.828 | 0.279 | 0.200 | 0.213 | 0.209 |
| Tm.Veg | 0.973 | 0.005 | 0.043 | 0.005 | 0.015 |
| Tm.NVeg | 0.935 | 0.197 | 0.134 | 0.034 | 0.005 |
| Tn.Veg | 0.942 | 0.196 | 0.069 | 0.159 | 0.050 |
| Tn.NVeg | 0.848 | 0.207 | 0.170 | 0.220 | 0.017 |
| Tx.Veg | 0.888 | 0.115 | 0.100 | 0.053 | 0.160 |
| Tx.NVeg | 0.901 | 0.122 | 0.035 | 0.087 | 0.063 |
| P.Veg | 0.404 | 0.829 | 0.248 | 0.158 | 0.083 |
| P.NVeg | 0.107 | 0.854 | 0.415 | 0.103 | 0.093 |
| **Eigenval** | 9.56 | 2.77 | 1.03 | 0.82 | 0.63 |
| **Variance** | 59.73 | 17.30 | 6.44 | 5.15 | 3.92 |
| **Cum.var** | 59.73 | 77.02 | 83.47 | 88.62 | 92.54 |

Table S3. Parameter estimates from multiple linear regressionswith the response variable CV_wp_ at tree ages 7, 8, 9, 10 and 15. Estimates of climate parameter TempPC and PrecPC from test site and population origin; respective p-values and adjusted coefficient of determination R² are given for each model.

|  | **AGE 7** | | **AGE 8** | | **AGE 9** | | **AGE 10** | | **AGE 15** | |
| --- | --- | --- | --- | --- | --- | --- | --- | --- | --- | --- |
|  | **Estimate** | **P** | **Estimate** | **P** | **Estimate** | **P** | **Estimate** | **P** | **Estimate** | **P** |
| **(Intercept)** | 0.233 | 0.000 | 0.232 | 0.000 | 0.251 | 0.000 | 0.272 | 0.000 | 0.290 | 0.000 |
| **TempPC_S** | -0.002 | 0.000 | 0.000 | 0.858 | 0.002 | 0.001 | 0.002 | 0.000 | -0.012 | 0.000 |
| **TempPC_P** | -0.002 | 0.000 | -0.003 | 0.000 | -0.003 | 0.000 | -0.002 | 0.000 | -0.002 | 0.033 |
| **PrecPC_S** | 0.004 | 0.000 | 0.003 | 0.000 | 0.004 | 0.000 | 0.006 | 0.000 | 0.013 | 0.000 |
| **PrecPC_P** | 0.001 | 0.399 | 0.001 | 0.141 | 0.002 | 0.043 | 0.002 | 0.025 | 0.002 | 0.075 |
| **adj. R²** | 0.13 |  | 0.077 |  | 0.057 |  | 0.062 |  | 0.424 |  |
|  |  |  |  |  |  |  |  |  |  |  |

Table S4. Parameter estimates of mixture model analyses for each combination of site X population subsets and for TempPC and PrecPC. *Lambda* gives the portion of attributed data to each component, *mu* is the component mean and *sigma* is the standard deviation.

|  | *lambda* | | *mu* | | *sigma* | |
| --- | --- | --- | --- | --- | --- | --- |
|  | Comp1 | Comp2 | Comp1 | Comp2 | Comp1 | Comp2 |
| **TempPC** |  |  |  |  |  |  |
| P1/S1 | 0.87 | 0.13 | 124.28 | 252.17 | 46.82 | 46.82 |
| P1/S2 | 0.63 | 0.37 | 225.31 | 379.68 | 78.90 | 78.90 |
| P1/S3 | 0.67 | 0.33 | 281.70 | 461.40 | 92.76 | 92.76 |
|  |  |  |  |  |  |  |
| P2/S1 | 0.86 | 0.14 | 128.10 | 264.32 | 45.50 | 45.50 |
| P2/S2 | 0.56 | 0.44 | 224.80 | 395.38 | 81.94 | 81.94 |
| P2/S3 | 0.47 | 0.53 | 278.26 | 488.37 | 94.98 | 94.98 |
|  |  |  |  |  |  |  |
| P3/S1 | 0.85 | 0.15 | 134.48 | 262.04 | 43.53 | 43.53 |
| P3/S2 | 0.44 | 0.56 | 207.78 | 389.28 | 79.82 | 79.82 |
| P3/S3 | 0.46 | 0.54 | 269.85 | 507.40 | 92.20 | 92.20 |
|  |  |  |  |  |  |  |
| **PrecPC** |  |  |  |  |  |  |
| P1/S1 | 0.43 | 0.57 | 205.90 | 463.74 | 90.23 | 90.23 |
| P1/S2 | 0.66 | 0.34 | 255.42 | 565.39 | 114.59 | 114.59 |
| P1/S3 | 0.51 | 0.49 | 202.62 | 390.43 | 65.02 | 65.02 |
|  |  |  |  |  |  |  |
| P2/S1 | 0.57 | 0.43 | 206.41 | 451.13 | 87.73 | 87.73 |
| P2/S2 | 0.84 | 0.16 | 247.23 | 530.57 | 115.44 | 115.44 |
| P2/S3 | 0.73 | 0.27 | 164.34 | 352.92 | 68.45 | 68.45 |
|  |  |  |  |  |  |  |
| P3/S1 | 0.57 | 0.43 | 188.65 | 433.71 | 84.08 | 84.08 |
| P3/S2 | 0.82 | 0.18 | 240.41 | 524.38 | 109.73 | 109.73 |
| P3/S3 | 0.77 | 0.23 | 167.94 | 365.95 | 67.50 | 67.50 |

**Table S5.** Parameter estimates of multiple regression analyses, predicting CV_wp_ by climate parameters TempPC and PrecPC at test sites (*_S) and population origins (*_P) using two different methods of data weighting. As with weight#1 the model adjusted R² is 0.4963 (F-statistic: 142.1 on 4 and 814 degrees of freedom), with weight#2 the adjusted R² is 0.4083 (F-statistic: 142.1 on 4 and 814 degrees of freedom).

| **Weighting** |  | **Estimate** | **Std. Error** | **t-value** | **Pr(>\|t\|)** |  |
| --- | --- | --- | --- | --- | --- | --- |
| **Weight#1** | **(Intercept)** | 0.2973 | 0.0024 | 123060 | < 0.001 | *** |
|  | **TempPC_S** | -0.0124 | 0.0006 | -21626 | < 0.001 | *** |
|  | **TempPC_P** | -0.0016 | 0.0006 | -2869 | 0.0042 | ** |
|  | **PrecPC_S** | 0.0130 | 0.0014 | 9391 | < 0.001 | *** |
|  | **PrecPC_P** | 0.0006 | 0.0014 | 396 | 0.6919 |  |
| **Weight#2** | **(Intercept)** | 0,2887 | 0,0024 | 121869 | 0,0000 | *** |
|  | **TempPC_S** | -0,0109 | 0,0007 | -15067 | 0,0000 | *** |
|  | **TempPC_P** | -0,0021 | 0,0008 | -2684 | 0,0074 | ** |
|  | **PrecPC_S** | 0,0138 | 0,0013 | 10993 | 0,0000 | *** |
|  | **PrecPC_P** | 0,0031 | 0,0014 | 2226 | 0,0263 | * |
